# Muscle and Tendon Morphology in Early-Adolescent Athletes and Untrained Peers

**DOI:** 10.3389/fphys.2020.01029

**Published:** 2020-08-21

**Authors:** Falk Mersmann, Gunnar Laube, Sebastian Bohm, Adamantios Arampatzis

**Affiliations:** ^1^Department of Training and Movement Sciences, Humboldt-Universität zu Berlin, Berlin, Germany; ^2^Berlin School of Movement Science, Humboldt-Universität zu Berlin, Berlin, Germany

**Keywords:** maturation, exercise, adaptation, imbalance, hypertrophy, KINGS project

## Abstract

Adolescent athletes can feature significantly greater muscle strength and tendon stiffness compared to untrained peers. However, to date, it is widely unclear if radial muscle and tendon hypertrophy may contribute to loading-induced adaptation at this stage of maturation. The present study compares the morphology of the vastus lateralis (VL) and the patellar tendon between early-adolescent athletes and untrained peers. In 14 male elite athletes (A) and 10 untrained controls (UC; 12–14 years of age), the VL was reconstructed from full muscle segmentations of magnetic resonance imaging (MRI) sequences and ultrasound imaging was used to measure VL fascicle length and pennation angle. The physiological cross-sectional area (PCSA) of the VL was calculated by dividing muscle volume by fascicle length. The cross-sectional area (CSA) of the patellar tendon was measured over its length based on MRI segmentations as well. Considering body mass as covariate in the analysis, there were no significant differences between groups considering the VL anatomical cross-sectional area (ACSA) over its length or maximum ACSA (UC: 24.0 ± 8.3 cm^2^, A: 28.1 ± 5.3 cm^2^, *p* > 0.05), yet athletes had significantly greater VL volume (UC: 440 ± 147 cm^3^, A: 589 ± 121 cm^3^), PCSA (UC: 31 ± 9 cm^2^, A: 46 ± 9 cm^2^), pennation angle (UC: 8.2 ± 1.4°, A: 10.1 ± 1.3°), and average patellar tendon CSA (UC: 1.01 ± 0.18 cm^2^, A: 1.21 ± 0.18 cm^2^) compared to the untrained peers (*p* < 0.05). However, the ratio of average tendon CSA to VL PCSA was significantly lower in athletes (UC: 3.4 ± 0.1%, A: 2.7 ± 0.5%; *p* < 0.05). When inferring effects of athletic training based on the observed differences between groups, these results suggest that both muscle and tendon of the knee extensors respond to athletic training with radial growth. However, the effect seems to be stronger in the muscle compared to the tendon, with an increase of pennation angle contributing to the marked increase of muscle PCSA. A disproportionate response to athletic training might be associated with imbalances of muscle strength and tendon stiffness and could have implications for the disposition towards tendon overuse injury.

## Introduction

Muscles and tendons interact and regulate the temporal characteristics of forces needed for the execution and control of movements. While the contractile element is able to generate forces and mechanical work, the elasticity of the connective tissue enables the storage and release of strain energy and affects the operating conditions of the muscle with regard to its force-length-velocity relationship (Kawakami and Fukunaga, 2006; Roberts and Azizi, 2011; Bohm et al., 2018, 2019). Both tissues are mechanosensitive and adapt to their mechanical environment. In muscle, an increase of habitual loading – for example, due to exercise – can trigger changes in its activation, physiological cross-sectional area (PCSA; including potential fiber type-specific hypertrophy), and specific tension, contributing to an increased capacity of the neuromuscular system to generate force (Aagaard et al., 2001; Folland and Williams, 2007; Erskine et al., 2010). Tendons can adapt to higher mechanical loading with an increase of stiffness (Arampatzis et al., 2007, 2010; Kongsgaard et al., 2007; Bohm et al., 2014), which is the capacity of the tendon to resist axial deformation upon force application. At a given rest length of the tendon, tendon stiffness depends on its cross-sectional area (CSA) and material properties, while the latter seems to change relatively early in a training process and the former is considered a long-term-mechanism to increased tendon stiffness (Bohm et al., 2015).

As most of the research on human muscle-tendon adaptation has been conducted on adults, it is less clear how muscles and, especially, tendons respond to increased mechanical loading during maturation. While there is no doubt that muscle strength can be increased by means of training already at prepubescent age, there is an ongoing debate about at which stage of maturity muscle hypertrophy has a relevant contribution. A prominent view is that the potential for exercise-induced muscle hypertrophy is low until the adolescent growth spurt (Lloyd et al., 2015). It should be noted, however, that only few studies investigated differences in muscle size following intervention or between trained and untrained cohorts with elaborate measures (i.e., magnet resonance or computerized tomography imaging; Ramsay et al., 1990; Daly et al., 2004; Granacher et al., 2011; Greene et al., 2012; Sanchis-Moysi et al., 2012) and none of those addressed long-term effects of training on lower extremity muscles in early adolescence (around the adolescent growth spurt). Recently, we found greater vastus lateralis (VL) thickness in early adolescent athletes compared to untrained controls (Charcharis et al., 2019), indicating training-induced muscle growth. However, the morphological determinant of radial muscle growth is the PCSA, which has not been measured in any study on lower limb muscle hypertrophy during maturation.

Data on the effects of loading on tendon morphology during maturation are essentially lacking. Waugh et al. (2014) provided evidence that, in preadolescent children, Achilles tendon stiffness can increase in response to specific mechanical stimulation that can be considered optimal for stimulating tendon adaptation. No indications for tendon hypertrophy were found in that study, though it should be noted that the accuracy of the applied ultrasound-based assessment of tendon CSA has been called into question by other work (Ekizos et al., 2013; Bohm et al., 2016; Kruse et al., 2016). Moreover, athletic activity that is mainly characterized by plyometric loading does not seem to significantly increase tendon stiffness in preadolescent children (Pentidis et al., 2019), while both early‐ (Charcharis et al., 2019) as well as late-adolescent athletes (Mersmann et al., 2017c) have stiffer tendons compared to untrained peers, even without specific tendon training. It is, however, unknown if tendon hypertrophy contributes to a loading-induced increase in stiffness in that age. As the half-life of collagen is estimated to be almost tenfold higher compared to muscle proteins (Lundholm et al., 1981; Thorpe et al., 2010) and basal tendon tissue turnover seems to essentially come to a halt at the end of adolescence (Heinemeier et al., 2013; Zhang et al., 2020), a morphological response of tendons to exercise can be expected during adolescence. It could well be that – similar to bone (Bachrach, 2001) – the acquisition of tendon tissue mass during growth might be a determinant of tendon strength throughout life. Identifying periods of pronounced tendon plasticity during maturation might help to formulate recommendations on when to target tendon adaptation in the long-term athletic development (Lloyd et al., 2015).

As the effects of athletic training on muscle and tendon morphology during early-adolescence are widely unknown, the present study investigates the morphology of the VL – as representative for the quadriceps femoris – including its PCSA and the patellar tendon CSA, due to the important contribution of the knee extensor muscle-tendon unit to movement performance and the susceptibility of the patellar tendon to overuse injury (Lian et al., 2005; Zwerver et al., 2011; Simpson et al., 2016; Nikolaidou et al., 2017). Using a cross-sectional design, comparing early-adolescent athletes with untrained peers, we hypothesized to find significantly greater VL PCSA and tendon CSA in athletes.

## Materials and Methods

### Participants and Experimental Design

Fourteen male adolescent athletes (handball and basketball) in the age of 12–14 years and 10 similar-aged controls volunteered to participate in the present study. The weekly training duration (with regard to the last 6 month and not considering competitions or school-based sports) of the handball athletes (*n* = 10) was about 10 h, including a total of approximately 2 hours of strength training. The basketball athletes (*n* = 4) trained about 8 h a week, including a total of about 80 min of strength-oriented exercises. Both groups only performed body-weight based strength exercises and did not participate in machine-based resistance training. All athletes were engaging in sport-specific training for at least 3 years at the time of measurement. For inclusion in the control group, a maximum duration of 2 h of leisure sport activity was allowed. Exclusion criteria were musculoskeletal diseases that affect muscle or tendon morphology (e.g., muscular dystrophy or patellar tendinopathy). All participants and respective legal guardians gave written informed consent to the experimental procedures, which were approved by the ethics committee of the Charité, University Medicine Berlin (EA2/076/15) and carried out in accordance with the declaration of Helsinki. VL architecture and muscle and patellar tendon morphology were assessed using ultrasound and magnetic resonance imaging (MRI) of the dominant leg, respectively. Leg dominance refers to the preference for kicking a ball. Since the athletes took part in a larger study featuring a more extensive data acquisition, the ultrasound and MRI data were captured on 2 separate days within 1 week in different facilities. In the control group, a portable ultrasound system was used to measure muscle architecture in conjunction with the MRI scanning. All participants were asked to refrain from vigorous physical activity the day before and on the day of the measurements.

### Assessment of Vastus Lateralis Architecture

Fascicle length and pennation angle of the VL were determined using ultrasonography. A linear ultrasound probe was placed over the mid-portion of the muscle belly at about 60% thigh length (from proximal to distal), which is the approximate location of the maximum anatomical cross-sectional area (ACSA; Mersmann et al., 2015). The knee angle was set to 60°, which is likely close to the optimum angle for force production in the knee joint (Herzog et al., 1990), and was controlled with a goniometer. The ultrasound device used on the athletes featured a 10 cm linear probe (7.5 MHz; My Lab60; Esaote, Genova, Italy), while a mobile ultrasound system with a 6-cm probe (8.0 MHz; Telemed Echoblaster 128; Telemed, Vilnius, Lithuania) was used in the control group. The root mean square differences in the measurement of fascicle length between the two devices were determined in a pilot study (young adults; *n* = 11) and were 7.6 mm with an intraclass correlation coefficient for absolute agreement and single measures of 0.912. The ultrasound images (five per participant) were analyzed using a custom written MATLAB interface (version R2016a; MathWorks, Natick, MA, USA). The upper and deeper aponeuroses were defined by manually setting reference points along the aponeuroses that were approximated by a linear least squares fitting. Subsequently, features of multiple fascicles were tracked automatically using the algorithm of Marzilger et al. (2018) and complemented manually when less than 10 fascicle fragments were automatically detected. A reference fascicle was calculated based on the average inclination of the fascicle portions, and fascicle length was measured as the Euclidian distance between intersection points of the reference fascicle and the two aponeuroses. The average fascicle length and pennation angle of the five images were fed into the statistical analysis. The pennation angle refers to the angle between the reference fascicle and the deeper aponeurosis.

### Assessment of Vastus Lateralis Morphology

Vastus lateralis muscle morphology was measured using an open 0.25 Tesla MRI scanner (G-Scan, Esaote, Genova, Italy). The participants were lying supine with the knee flexed to 10° (0° = full extension) to align the thigh with the longitudinal axis of the scanner. Since the scanner had a small field of view, the thigh was separated into three sections from the greater trochanter to the lateral epicondyle (proximal, medial, and distal), which were captured in consecutive Turbo 3D T1 weighted sequences (TE: 16 ms, TR: 39 ms, slice thickness: 3.1 mm, no gaps). The boundaries of the thigh-sections were marked with lines of fish-oil capsules, which were fixed to the skin using self-adhesive tape to avoid any dislocation during the repositioning of the leg within the field of view. The contours of the VL and fish-oil capsules in the respective MRI sequences of the proximal, medial, and distal thigh-sections were then manually outlined using Osirix (version 7.0, Pixmeo, Geneva, Swiss). The lines of fish-oil capsules that were captured and segmented in two adjacent sections were used to reassemble the three partial volumes to the full muscle. The custom-written MATLAB algorithm used for this procedure is described in more detail elsewhere (Marzilger et al., 2019). The average error associated with the reconstruction from multiple sequences is estimated to range between 1 and 2% (Marzilger et al., 2019). From the reconstructed muscle, we examined muscle volume, maximal ACSA (ACSA_max_), and ACSA in 10%-intervals along the muscle length. The PCSA was calculated as quotient of muscle volume and fascicle length (Lieber and Fridén, 2000).

### Assessment of Tendon Morphology

The CSA of the patellar tendon was determined based on segmentations of MRI sequences as well. 3D HYCE (GR) sequences (10 ms repetition time, 5 ms excitation time, 80° flip angle, 3 mm slice thickness, and one excitation) of the knee joint were recorded using the same scanner and subject positioning as described above. The boundaries of the patellar tendon were segmented in OsiriX between the distal apex of the patellar and deep insertion at the tibial tuberosity. As recommended by Couppé et al. (2013), we applied the NIH color scale during the segmentation to facilitate the accurate tracing the contours of the tendon. Since it is barely possible to perfectly align the longitudinal axis of the tendon with the longitudinal axis of the MRI scanner, simple transverse plane segmentations would overestimate the CSA. For this reason, the digitized patellar tendon CSAs were transformed orthogonal to the line of action of the patellar tendon, which in turn was defined as the line of best fit through the geometrical centers of the respective CSAs (Bohm et al., 2016). The CSAs were then analyzed in 10%-intervals along the tendon length.

### Statistics

Normality of the data was analyzed by means of the Shapiro-Wilk test and was given in all parameters (or respective standardized residuals where applicable) except age and VL muscle ACSA in some length intervals. Baseline differences in age were analyzed with the Mann-Whitney U test and anthropometry using the Student’s *t*-test for independent samples. VL pennation angle, fascicle length, ACSA_max_ and PCSA, and average patellar tendon CSA were analyzed using a one-way analysis of variance (ANOVA). Muscle and tendon CSAs over their lengths were analyzed in 10%-intervals in a repeated measures ANOVA with the length-intervals as within-subject factor. The ANOVA model was used due to its robustness considering the type of data distribution (Schmider et al., 2010). The Greenhouse-Geisser correction was applied if the assumption of sphericity was violated. As body mass and height differed notably between groups, we used covariates in the statistical analysis where applicable and indicated their use as subscript of the reported values of *p* in the results section (BM: body mass for VL and patellar tendon CSAs, as well as VL volume and pennation angle; FL: femur length for fascicle length). Further, we analyzed the association of VL PCSA and patellar tendon CSA as well as between the PCSA and pennation angle using the Pearson correlation coefficient (*r*) and calculated the explained variance as *r*^2^. The alpha level for all statistical tests was set to 0.05. The effect size *f* was calculated based on either Cohens *d* or partial eta squared (depending on the type of original test) and categorized as either small (0.1 ≤ *f* < 0.25), medium (0.25 ≤ *f* < 0.5), or large (*f* ≥ 0.5; Cohen, 2013).

## Results

**Figure 1:**
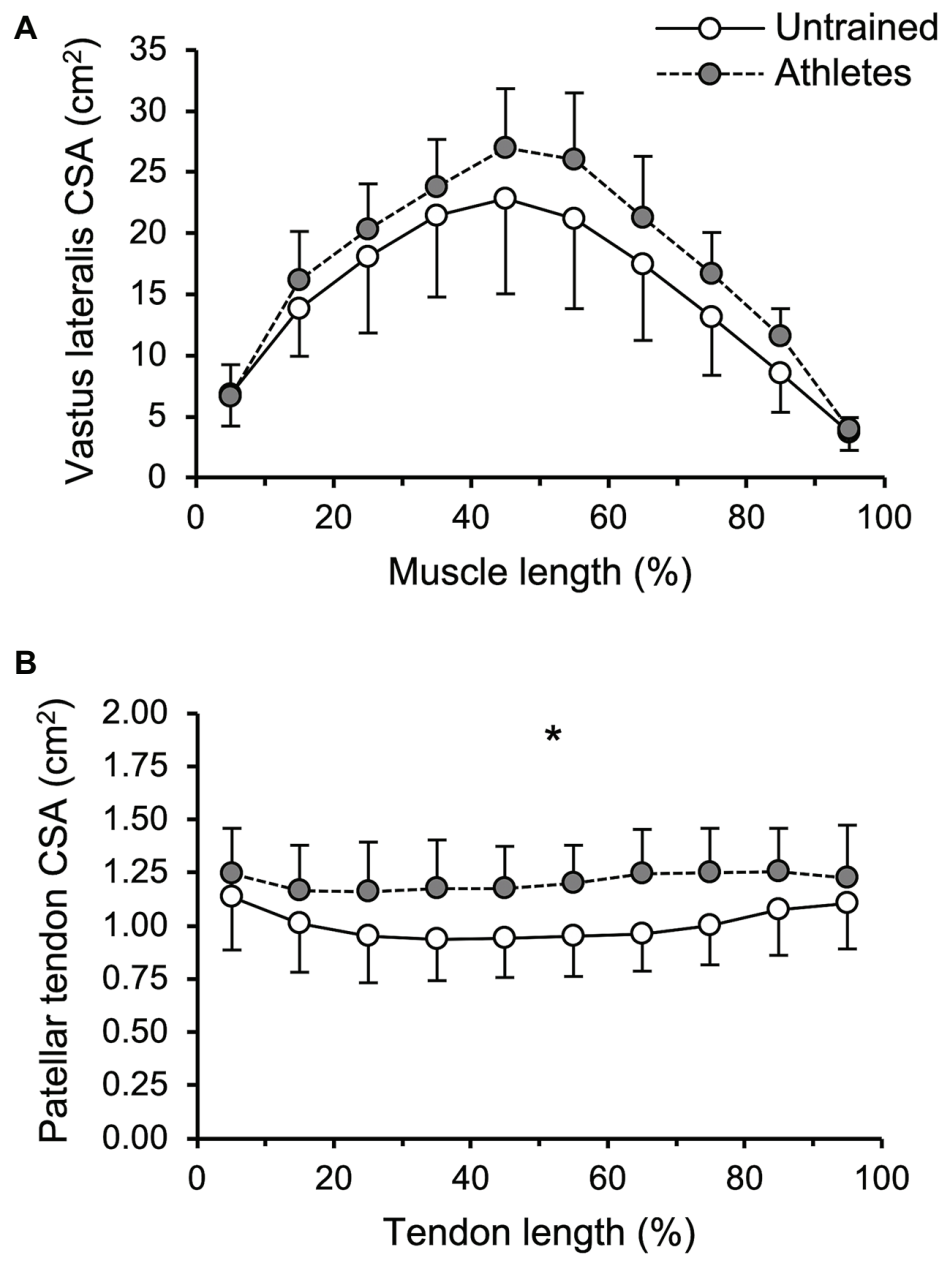
**(A)** Vastus lateralis (VL) anatomical cross-sectional area and **(B)** patellar tendon cross-sectional area (CSA) of adolescent athletes (gray; *n* = 14) and untrained controls (white; *n* = 10) as a function of muscle or tendon length. The values represent average values ± SD of 10%-intervals (i.e., 0–10%, …), respectively. Note that body mass was used as covariate in the statistical analysis. ^*^Significant main effect of group (*p* < 0.05).

**Figure 2:**
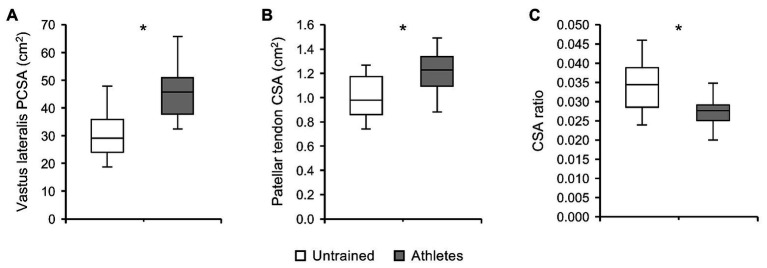
(A) Vastus lateralis physiological cross-sectional area (PCSA), **(B)** patellar tendon CSA, and **(C)** ratio of average tendon CSA to VL PCSA of adolescent athletes (gray; *n* = 14) and untrained controls (white; *n* = 10). Note that body mass was used as covariate in the statistical analysis of VL PCSA and patellar tendon CSA. ^*^Significant difference (*p* < 0.05).

**Figure 3:**
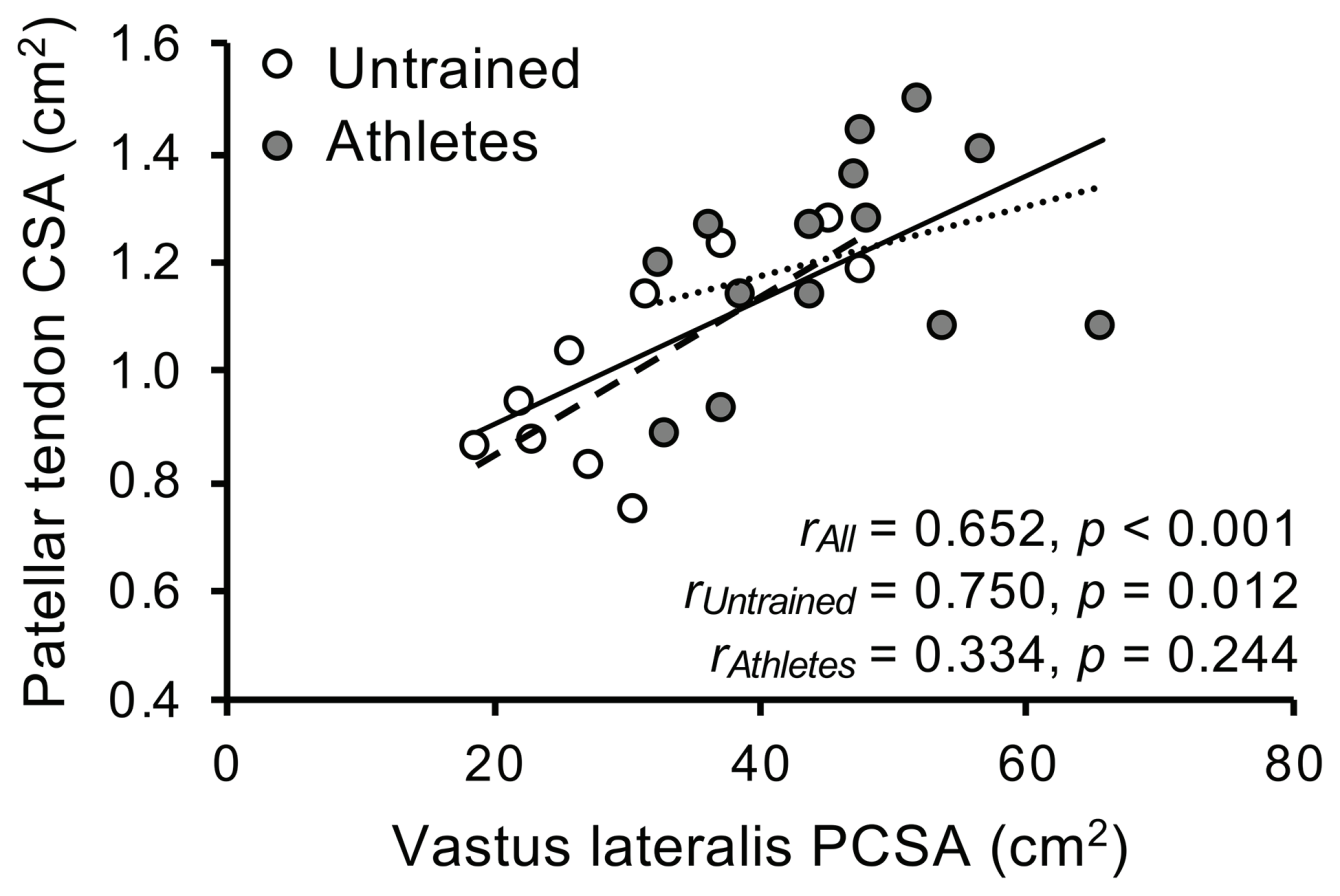
Association of vastus lateralis physiological cross-sectional area and patellar tendon cross-sectional area (CSA) in adolescent athletes (gray; *n* = 14) and untrained controls (white; *n* = 10).

There was no significant difference in calendar age between groups (athletes: 13.9 ± 0.5 years, untrained: 13.4 ± 1.0 years; *p* = 0.341; *f* = 0.32). The athletes were significantly taller compared to untrained controls (athletes: 175 ± 9 cm, untrained: 164 ± 13 cm; *p* = 0.02; *f* = 0.5), yet the differences in body mass did not reach significance (athletes: 64.4 ± 12.5 kg, untrained: 55.9 ± 12.2 kg; *p* = 0.112; *f* = 0.34). With body mass as a covariate, there was no significant effect of group (*p_BM_* = 0.736; *f* = 0.08) or group by length interaction (*p_BM_* = 0.402; *f* = 0.22) on VL ACSA (Figure 1). Vastus lateralis PCSA (*p_BM_* = 0.007; *f* = 0.66; Figure 2) and volume (*p_BM_* = 0.044; *f* = 0.47; Table 1) were significantly greater in athletes than untrained controls, while muscle ACSA_max_ did not differ significantly between groups (*p_BM_* = 0.917; *f* = 0.03; Table 1). Vastus lateralis pennation angle was greater in athletes (*p_BM_* = 0.006; *f* = 0.66), while fascicle length did not differ significantly between groups (*p_FL_* = 0.393; *f* = 0.19; Table 1). Patellar tendon CSA was significantly greater in athletes compared to untrained controls (*p_BM_* = 0.049; *f* = 0.44) without significant group by length interaction (*p_BM_* = 0.263; *f* = 0.25; Figure 1). Therefore, average tendon CSA was also significantly greater in athletes (*p_BM_* = 0.049; *f* = 0.44; Figure 2). The ratio of average patellar tendon CSA to VL PCSA was, however, significantly lower in athletes than controls (*p* = 0.016; *f* = 0.56; Figure 2). Further, there was a significant correlation between VL PCSA and tendon CSA for both groups combined (*r* = 0.652, *p* < 0.001; Figure 3). However, when analyzed separately, the correlation was significant in controls (*r* = 0.750, *p* = 0.012) and not in athletes (*r* = 0.334, *p* = 0.224). In addition, there was a significant correlation between the VL PCSA and pennation angle for both groups combined (*r* = 0.521; *p* = 0.009).

**Table 1:** Parameters of vastus lateralis (VL) morphology and architecture in early adolescent athletes and untrained controls.

	Untrained (*n* = 10)	Athletes (*n* = 14)
ACSA_max_ (cm^2^)^BM^	24.0 ± 8.3	28.1 ± 5.3
Volume (cm^3^)^BM^^*^	440 ± 147	589 ± 121
Muscle length (cm)^*^	29.8 ± 3.2	34.0 ± 2.3
Fascicle length (cm)^FL^	14.3 ± 2.8	13.1 ± 2.3
Pennation angle (°)^BM^^*^	8.2 ± 1.4	10.1 ± 1.3

Values are means ± standard deviation. ACSA, anatomical cross-sectional area. Data were analyzed with ^BM^body mass or ^FL^femur length as covariate.

* Significantly different between groups (*p* < 0.05).

## Discussion

The present study investigated the effects of athletic training on knee extensor muscle and tendon morphology in early-adolescence by comparing elite athletes with untrained peers. With anthropometric differences considered as covariates in the statistical analysis, athletes did not demonstrate significantly greater VL ACSA_max_ than controls but a markedly larger PCSA, volume, and a significantly greater pennation angle. The patellar tendon CSA was also significantly greater in athletes and, thus, our main hypotheses were confirmed. However, the ratio of tendon CSA to muscle PCSA as well as the association between them was lower in athletes compared to controls, which might indicate an imbalance of muscle and tendon in the radial growth-response to athletic training in that age.

The present study provides evidence of marked differences in knee extensor muscle and tendon morphology between early-adolescent athletes and untrained peers, indicating loading-induced hypertrophy of both tissues. Compared to untrained peers and under consideration of body mass, the athletes had a significantly larger VL PCSA and patellar tendon CSA. As the basal levels of muscle-anabolic sex hormones are low before puberty (Murray and Clayton, 2013), the morphological response of muscles to mechanical loading is considered to be negligible in children (Myer et al., 2011). Not later than with the onset of adolescence, however, the responsiveness of skeletal muscles to loading in terms of morphological adaptation might change in conjunction with its endocrine environment. We recently found greater VL thickness in early-adolescent athletes compared to untrained peers (Charcharis et al., 2019). The present study extends these findings to the muscles PCSA, which is the main morphological determinant of radial muscle growth and number of sarcomeres in parallel (Haxton, 1944). We found a large effect of group (*f* = 0.66) with significantly greater PCSAs of the VL in the athletes compared to untrained controls, which may suggest a marked radial adaptation of the muscle to athletic training. Since the ACSA_max_ did not differ significantly between groups, this difference was likely influenced by the greater pennation angle of the muscle, which correlated significantly with the PCSA and explained 27% of its variance. It has been demonstrated earlier in adults that architectural remodeling enables the loading-induced changes of muscle PCSA to exceed those of the ACSA (Aagaard et al., 2001). The present findings and earlier studies comparing mid-adolescent volleyball athletes with untrained peers (Mersmann et al., 2016, 2017c) suggest that an increase of pennation angle contributes to radial muscle adaptation during maturation. This underlines the importance of considering muscle architecture when investigating muscle plasticity and adds to the uncertainty about muscle hypertrophy in prepubescent children, since muscle architecture has not been investigated in the respective studies on pennate muscles (Ramsay et al., 1990; Granacher et al., 2011).

Similar as in muscles, the morphology of tendons also seems to adapt to mechanical loading during maturation. Even with body mass accounted for, we found significantly greater patellar tendon CSA in early-adolescent athletes compared to the untrained group. As there was no group by length interaction, which indicates hypertrophy of the whole tendon as opposed to only regional differences e.g., due to pathological swelling, we are confident that our data indicate a physiological (adaptive) effect of athletic training on tendon morphology. Therefore, it seems likely that tendon hypertrophy may have contributed to the higher tendon stiffness we observed in early-adolescent athletes compared to untrained peers in an earlier study (Charcharis et al., 2019). Compared to muscle PCSA, however, the differences between the athletes and untrained controls were lower (i.e., medium effect; *f* = 0.46) and less consistent. Further, the ratio of tendon CSA to muscle PCSA was significantly lower in athletes and the correlation of the two morphological parameters was only significant in controls. This might indicate that there is a disproportionate and less coordinated change of muscle PCSA and tendon CSA under the two-fold stimulus of athletic training and maturation. In an earlier study, we found that the VL PCSA of mid-adolescents volleyball athletes was already similar to adult athletes, while the patellar tendon CSA was still markedly smaller (Mersmann et al., 2014). In a subsequent longitudinal study over two years, the patellar tendons of those adolescent athletes demonstrated remarkable radial growth of about 27%, which can be considered a physiological adaptation regarding the unchanged elastic modulus of the tendon (Mersmann et al., 2017b). It is possible that the capacity of the tendon to adapt to mechanical loading with an increase of its CSA is rather low until body growth rate declines. Mid‐ to late-adolescence could then be a period where tendon tissue mass can be acquired effectively by means of training until tissue turnover greatly reduces (Heinemeier et al., 2013; Zhang et al., 2020). Early‐ to mid-adolescence on the other hand may be considered a critical period for the tendon, as the morphological adaptation to loading seems to occur at a lower rate compared to the muscle. This might contribute to the imbalances of muscle strength and tendon stiffness, which can increase the mechanical demand of the tendon and might associate to the increasing risk of tendon overuse injury with the onset of adolescence (Simpson et al., 2016; Mersmann et al., 2017a, 2019). However, more long-term longitudinal studies during adolescence are needed to support these assumptions. Similarly, it remains to be shown to what extend the findings of the present study transfer to athletes from different sports. It seems reasonable to assume that activities that induce great mechanical and metabolic stress for the muscle due to high force and power output have the potential to induce muscle hypertrophy (Schoenfeld, 2013). Similarly, high forces or, more specifically, high magnitudes of tendon strain, are crucial for tendon adaptation as well (Arampatzis et al., 2007; Kongsgaard et al., 2007). However, short strain durations as in plyometric loading may not lead to an effective tendon stimulation (Burgess et al., 2007; Fouré et al., 2009; Houghton et al., 2013; Bohm et al., 2014), which may contribute to imbalances of muscle and tendon adaptation in sports that are characterized by a high frequency of jumps or change-of-direction movements (Mersmann et al., 2017a).

A common challenge in studies with adolescents is the potential discrepancy between calendar and biological age. However, the assessment of skeletal age involves exposure to radiation and the accuracy of grading the secondary sex characteristics is rather low (Schlossberger et al., 1992; Taylor et al., 2001; Slough et al., 2013). Maturity can roughly be estimated based on anthropometric data (Mirwald et al., 2002; Moore et al., 2015), yet these predictions cannot account for the considerable variation in anthropometry at a similar stage of maturity, which is a particular problem with athletes from sports with body height as a selection criterion. However, data on the Achilles tendon suggest that potential effects of differing maturity on tendon properties is mainly attributed to (maturation-related) differences in body mass (Waugh et al., 2012). As body mass was used as a covariate in the present study, we are confident that the lack of an adequate control for biological age does not affect our conclusions. Nevertheless, the possibility that mass-independent effects of biological maturity and hormone status may have influenced our results cannot be discarded completely. An additional potential limitation of the study is the use of two ultrasound systems with different fields of view for the two groups. Considering the length of the VL fascicles, ~58 and ~23% of the calculated reference fascicles exceeded the field of view in the untrained control and athlete group, respectively. However, the agreement when scanning with both systems in the pilot study was fairly good and suggests a measurement error of about 5%. Even when systematically decreasing the VL fascicle length of the untrained control group by 5% (thus increasing PCSA), the differences between groups in VL PCSA remained significant (*p* = 0.019). We are also convinced that the differences found in PCSA are representative for the whole muscle despite that only the mid-region of the muscle was considered in the assessment of fascicle length.

In conclusion, the present study provides evidence that athletes feature greater VL PCSA and patellar tendon CSA already in early-adolescence compared to untrained peers, indicating loading-induced hypertrophy in both tissues. However, there seems to be a disproportionate adaptation to athletic training, leading to a lower ratio of tendon to muscle CSA in athletes. This imbalance might contribute to the mismatch of muscle strength and tendon stiffness that has been reported earlier (Mersmann et al., 2017c; Charcharis et al., 2019), with potential implications for the risk of tendon injury (Simpson et al., 2016; Mersmann et al., 2019). Future research might clarify if mid‐ to late-adolescence is a period of pronounced tendon plasticity and if tendon adaptation can be promoted earlier during maturation by means of specific interventions.

## Data Availability Statement

The raw data supporting the conclusions of this article will be made available by the authors, without undue reservation.

## Ethics Statement

The studies involving human participants were reviewed and approved by Ethics Committee of the Humboldt-Universität zu Berlin (EA2/076/15). Written informed consent to participate in this study was provided by the participants’ legal guardian/next of kin.

## Author Contributions

FM and AA conceived the experiment. FM and GL performed the experiments. FM and GL analyzed the data. FM and AA interpreted the data. FM, SB, and AA drafted the manuscript. GL made important intellectual contributions during revision. All authors approved the final version of the manuscript and agree to be accountable for the content of the work.

### Conflict of Interest

The authors declare that the research was conducted in the absence of any commercial or financial relationships that could be construed as a potential conflict of interest.

## References

[ref1] Aagaard P. Andersen J. L. Dyhre-Poulsen P. Leffers A. M. Wagner A. Magnusson S. P.. (2001). A mechanism for increased contractile strength of human pennate muscle in response to strength training: changes in muscle architecture. J. Physiol. 534, 613–623. 10.1111/j.1469-7793.2001.t01-1-00613.x, PMID: 11454977 PMC2278719

[ref2] Arampatzis A. Karamanidis K. Albracht K. (2007). Adaptational responses of the human Achilles tendon by modulation of the applied cyclic strain magnitude. J. Exp. Biol. 210, 2743–2753. 10.1242/jeb.003814, PMID: 17644689

[ref3] Arampatzis A. Peper A. Bierbaum S. Albracht K. (2010). Plasticity of human Achilles tendon mechanical and morphological properties in response to cyclic strain. J. Biomech. 43, 3073–3079. 10.1016/j.jbiomech.2010.08.014, PMID: 20863501

[ref4] Bachrach L. K. (2001). Acquisition of optimal bone mass in childhood and adolescence. Trends Endocrinol. Metab. 12, 22–28. 10.1016/S1043-2760(00)00336-2, PMID: 11137037

[ref5] Bohm S. Marzilger R. Mersmann F. Santuz A. Arampatzis A. (2018). Operating length and velocity of human vastus lateralis muscle during walking and running. Sci. Rep. 8:5066. 10.1038/s41598-018-23376-5, PMID: 29567999 PMC5864755

[ref6] Bohm S. Mersmann F. Arampatzis A. (2015). Human tendon adaptation in response to mechanical loading: a systematic review and meta-analysis of exercise intervention studies on healthy adults. Sports Med. Open 1:7. 10.1186/s40798-015-0009-9, PMID: 27747846 PMC4532714

[ref7] Bohm S. Mersmann F. Santuz A. Arampatzis A. (2019). The force-length-velocity potential of the human soleus muscle is related to the energetic cost of running. Proc. Biol. Sci. 286:20192560. 10.1098/rspb.2019.2560, PMID: 31847774 PMC6939913

[ref8] Bohm S. Mersmann F. Schroll A. Mäkitalo N. Arampatzis A. (2016). Insufficient accuracy of the ultrasound-based determination of Achilles tendon cross-sectional area. J. Biomech. 49, 2932–2937. 10.1016/j.jbiomech.2016.07.002, PMID: 27498950

[ref9] Bohm S. Mersmann F. Tettke M. Kraft M. Arampatzis A. (2014). Human Achilles tendon plasticity in response to cyclic strain: effect of rate and duration. J. Exp. Biol. 217, 4010–4017. 10.1242/jeb.112268, PMID: 25267851

[ref10] Burgess K. E. Connick M. J. Graham-Smith P. Pearson S. J. (2007). Plyometric vs. isometric training influences on tendon properties and muscle output. J. Strength Cond. Res. 21, 986–995. 10.1519/R-20235.1, PMID: 17685695

[ref11] Charcharis G. Mersmann F. Bohm S. Arampatzis A. (2019). Morphological and mechanical properties of the quadriceps femoris muscle-tendon unit from adolescence to adulthood: effects of age and athletic training. Front. Physiol. 10:1082. 10.3389/fphys.2019.01082, PMID: 31507446 PMC6718516

[ref12] Cohen J. (2013). Statistical power analysis for the behavioral sciences. New York: Acamemic Press.

[ref13] Couppé C. Svensson R. B. Sødring-Elbrønd V. Hansen P. Kjaer M. Magnusson S. P. (2013). Accuracy of MRI technique in measuring tendon cross-sectional area. Clin. Physiol. Funct. Imaging 34, 237–241. 10.1111/cpf.12086, PMID: 24119143

[ref14] Daly R. M. Saxon L. Turner C. H. Robling A. G. Bass S. L. (2004). The relationship between muscle size and bone geometry during growth and in response to exercise. Bone 34, 281–287. 10.1016/j.bone.2003.11.009, PMID: 14962806

[ref15] Ekizos A. Papatzika F. Charcharis G. Bohm S. Mersmann F. Arampatzis A. (2013). Ultrasound does not provide reliable results for the measurement of the patellar tendon cross sectional area. J. Electromyogr. Kinesiol. 23, 1278–1282. 10.1016/j.jelekin.2013.08.004, PMID: 24021864

[ref16] Erskine R. M. Jones D. A. Williams A. G. Stewart C. E. Degens H. (2010). Resistance training increases in vivo quadriceps femoris muscle specific tension in young men. Acta Physiol. 199, 83–89. 10.1111/j.1748-1716.2010.02085.x, PMID: 20102343

[ref17] Folland J. P. Williams A. G. (2007). The adaptations to strength training: morphological and neurological contributions to increased strength. Sports Med. 37, 145–168. 10.2165/00007256-200737020-00004, PMID: 17241104

[ref18] Fouré A. Nordez A. Guette M. Cornu C. (2009). Effects of plyometric training on passive stiffness of gastrocnemii and the musculo-articular complex of the ankle joint. Scand. J. Med. Sci. Sports 19, 811–818. 10.1111/j.1600-0838.2008.00853.x, PMID: 19508650

[ref19] Granacher U. Goesele A. Roggo K. Wischer T. Fischer S. Zuerny C.. (2011). Effects and mechanisms of strength training in children. Int. J. Sports Med. 32, 357–364. 10.1055/s-0031-1271677, PMID: 21380967

[ref20] Greene D. A. Naughton G. A. Bradshaw E. Moresi M. Ducher G. (2012). Mechanical loading with or without weight-bearing activity: influence on bone strength index in elite female adolescent athletes engaged in water polo, gymnastics, and track-and-field. J. Bone Miner. Metab. 30, 580–587. 10.1007/s00774-012-0360-6, PMID: 22614913

[ref21] Haxton H. A. (1944). Absolute muscle force in the ankle flexors of man. J. Physiol. 103, 267–273. 10.1113/jphysiol.1944.sp004075, PMID: 16991644 PMC1393493

[ref22] Heinemeier K. M. Schjerling P. Heinemeier J. Magnusson S. P. Kjaer M. (2013). Lack of tissue renewal in human adult Achilles tendon is revealed by nuclear bomb (14)C. FASEB J. 27, 2074–2079. 10.1096/fj.12-225599, PMID: 23401563 PMC3633810

[ref23] Herzog W. Abrahamse S. K. ter Keurs H. E. (1990). Theoretical determination of force-length relations of intact human skeletal muscles using the cross-bridge model. Pflugers Arch. 416, 113–119. 10.1007/BF00370231, PMID: 2352828

[ref24] Houghton L. A. Dawson B. T. Rubenson J. (2013). Effects of plyometric training on Achilles tendon properties and shuttle running during a simulated cricket batting innings. J. Strength Cond. Res. 27, 1036–1046. 10.1519/JSC.0b013e3182651e7a, PMID: 22739327

[ref25] Kawakami Y. Fukunaga T. (2006). New insights into in vivo human skeletal muscle function. Exerc. Sport Sci. Rev. 34, 16–21. 10.1097/00003677-200601000-00005, PMID: 16394810

[ref26] Kongsgaard M. Reitelseder S. Pedersen T. G. Holm L. Aagaard P. Kjaer M.. (2007). Region specific patellar tendon hypertrophy in humans following resistance training. Acta Physiol. 191, 111–121. 10.1111/j.1748-1716.2007.01714.x, PMID: 17524067

[ref27] Kruse A. Stafilidis S. Tilp M. (2016). Ultrasound and magnetic resonance imaging are not interchangeable to assess the Achilles tendon cross-sectional-area. Eur. J. Appl. Physiol. 117, 73–82. 10.1007/s00421-016-3500-1, PMID: 27838848 PMC5306331

[ref28] Lian O. B. Engebretsen L. Bahr R. (2005). Prevalence of jumper’s knee among elite athletes from different sports: a cross-sectional study. Am. J. Sports Med. 33, 561–567. 10.1177/0363546504270454, PMID: 15722279

[ref29] Lieber R. L. Fridén J. (2000). Functional and clinical significance of skeletal muscle architecture. Muscle Nerve 23, 1647–1666. 10.1002/1097-4598(200011)23:11<1647::AID-MUS1>3.0.CO;2-M, PMID: 11054744

[ref30] Lloyd R. S. Oliver J. L. Faigenbaum A. D. Howard R. De Ste Croix M. B. A. Williams C. A.. (2015). Long-term athletic development—part 1: a pathway for all youth. J. Strength Cond. Res. 29, 1439–1450. 10.1519/JSC.0000000000000756, PMID: 25486295

[ref31] Lundholm K. Edström S. Ekman L. Karlberg I. Walker P. Scherstén T. (1981). Protein degradation in human skeletal muscle tissue: the effect of insulin, leucine, amino acids and ions. Clin. Sci. 60, 319–326. 10.1042/cs0600319, PMID: 7016406

[ref32] Marzilger R. Legerlotz K. Panteli C. Bohm S. Arampatzis A. (2018). Reliability of a semi-automated algorithm for the vastus lateralis muscle architecture measurement based on ultrasound images. Eur. J. Appl. Physiol. 118, 291–301. 10.1007/s00421-017-3769-8, PMID: 29214464

[ref33] Marzilger R. Schroll A. Bohm S. Arampatzis A. (2019). Muscle volume reconstruction from several short magnetic resonance imaging sequences. J. Biomech. 84, 269–273. 10.1016/j.jbiomech.2018.12.038, PMID: 30655082

[ref34] Mersmann F. Bohm S. Arampatzis A. (2017a). Imbalances in the development of muscle and tendon as risk factor for tendinopathies in youth athletes: a review of current evidence and concepts of prevention. Front. Physiol. 8:987. 10.3389/fphys.2017.00987, PMID: 29249987 PMC5717808

[ref35] Mersmann F. Bohm S. Schroll A. Boeth H. Duda G. Arampatzis A. (2014). Evidence of imbalanced adaptation between muscle and tendon in adolescent athletes. Scand. J. Med. Sci. Sports 24, E283–E289. 10.1111/sms.12166, PMID: 24372566

[ref36] Mersmann F. Bohm S. Schroll A. Boeth H. Duda G. Arampatzis A. (2015). Muscle shape consistency and muscle volume prediction of thigh muscles. Scand. J. Med. Sci. Sports 25, e208–e213. 10.1111/sms.12285, PMID: 24975992

[ref37] Mersmann F. Bohm S. Schroll A. Boeth H. Duda G. N. Arampatzis A. (2017b). Muscle and tendon adaptation in adolescent athletes: a longitudinal study. Scand. J. Med. Sci. Sports 27, 75–82. 10.1111/sms.12631, PMID: 26644277

[ref38] Mersmann F. Bohm S. Schroll A. Marzilger R. Arampatzis A. (2016). Athletic training affects the uniformity of muscle and tendon adaptation during adolescence. J. Appl. Physiol. 121, 893–899. 10.1152/japplphysiol.00493.2016, PMID: 27586836

[ref39] Mersmann F. Charcharis G. Bohm S. Arampatzis A. (2017c). Muscle and tendon adaptation in adolescence: elite volleyball athletes compared to untrained boys and girls. Front. Physiol. 8:417. 10.3389/fphys.2017.00417, PMID: 28670285 PMC5472702

[ref40] Mersmann F. Pentidis N. Tsai M. S. Schroll A. Arampatzis A. (2019). Patellar tendon strain associates to tendon structural abnormalities in adolescent athletes. Front. Physiol. 10:963. 10.3389/fphys.2019.00963, PMID: 31427983 PMC6687848

[ref41] Mirwald R. L. Baxter-Jones A. D. G. Bailey D. A. Beunen G. P. (2002). An assessment of maturity from anthropometric measurements. Med. Sci. Sports Exerc. 34, 689–694. 10.1097/00005768-200204000-00020, PMID: 11932580

[ref42] Moore S. A. McKay H. A. Macdonald H. Nettlefold L. Baxter-Jones A. D. G. Cameron N.. (2015). Enhancing a somatic maturity prediction model. Med. Sci. Sports Exerc. 47, 1755–1764. 10.1249/MSS.0000000000000588, PMID: 25423445

[ref43] Murray P. G. Clayton P. E. (2013). Endocrine control of growth. Am. J. Med. Genet. C Semin. Med. Genet. 163, 76–85. 10.1002/ajmg.c.31357, PMID: 23613426

[ref44] Myer G. D. Faigenbaum A. D. Ford K. R. Best T. M. Bergeron M. F. Hewett T. E. (2011). When to initiate integrative neuromuscular training to reduce sports-related injuries and enhance health in youth? Curr. Sports Med. Rep. 10, 155–166. 10.1249/JSR.0b013e31821b1442, PMID: 21623307 PMC3105332

[ref45] Nikolaidou M. E. Marzilger R. Bohm S. Mersmann F. Arampatzis A. (2017). Operating length and velocity of human M. vastus lateralis fascicles during vertical jumping. R. Soc. Open Sci. 4:170185. 10.1098/rsos.170185, PMID: 28573027 PMC5451828

[ref46] Pentidis N. Mersmann F. Bohm S. Giannakou E. Aggelousis N. Arampatzis A. (2019). Triceps surae muscle-tendon unit properties in preadolescent children: a comparison of artistic gymnastic athletes and non-athletes. Front. Physiol. 10:615. 10.3389/fphys.2019.00615, PMID: 31164838 PMC6536691

[ref47] Ramsay J. A. Blimkie C. J. Smith K. Garner S. MacDougall J. D. Sale D. G. (1990). Strength training effects in prepubescent boys. Med. Sci. Sports Exerc. 22, 605–614. 10.1249/00005768-199010000-00011, PMID: 2233199

[ref48] Roberts T. J. Azizi E. (2011). Flexible mechanisms: the diverse roles of biological springs in vertebrate movement. J. Exp. Biol. 214, 353–361. 10.1242/jeb.038588, PMID: 21228194 PMC3020146

[ref49] Sanchis-Moysi J. Idoate F. Serrano-Sanchez J. A. Dorado C. Calbet J. A. L. (2012). Muscle hypertrophy in prepubescent tennis players: a segmentation MRI study. PLoS One 7:e33622. 10.1371/journal.pone.0033622, PMID: 22428074 PMC3302769

[ref50] Schlossberger N. M. Turner R. A. Irwin C. E. (1992). Validity of self-report of pubertal maturation in early adolescents. J. Adolesc. Health 13, 109–113. 10.1016/1054-139X(92)90075-M, PMID: 1627576

[ref51] Schmider E. Ziegler M. Danay E. Beyer L. Bühner M. (2010). Is it really robust? Methodology 6, 147–151. 10.1027/1614-2241/a000016

[ref52] Schoenfeld B. J. (2013). Potential mechanisms for a role of metabolic stress in hypertrophic adaptations to resistance training. Sports Med. 43, 179–194. 10.1007/s40279-013-0017-1, PMID: 23338987

[ref53] Simpson M. Rio E. Cook J. (2016). At what age do children and adolescents develop lower limb tendon pathology or tendinopathy? A systematic review and meta-analysis. Sports Med. 46, 545–557. 10.1007/s40279-015-0438-0, PMID: 26626072

[ref54] Slough J. M. Hennrikus W. Chang Y. (2013). Reliability of Tanner staging performed by orthopedic sports medicine surgeons. Med. Sci. Sports Exerc. 45, 1229–1234. 10.1249/MSS.0b013e318285c2f7, PMID: 23439412

[ref55] Taylor S. J. Whincup P. H. Hindmarsh P. C. Lampe F. Odoki K. Cook D. G. (2001). Performance of a new pubertal self-assessment questionnaire: a preliminary study. Paediatr. Perinat. Epidemiol. 15, 88–94. 10.1046/j.1365-3016.2001.00317.x, PMID: 11237120

[ref56] Thorpe C. T. Streeter I. Pinchbeck G. L. Goodship A. E. Clegg P. D. Birch H. L. (2010). Aspartic acid racemization and collagen degradation markers reveal an accumulation of damage in tendon collagen that is enhanced with aging. J. Biol. Chem. 285, 15674–15681. 10.1074/jbc.M109.077503, PMID: 20308077 PMC2871433

[ref57] Waugh C. M. Blazevich A. J. Fath F. Korff T. (2012). Age-related changes in mechanical properties of the Achilles tendon. J. Anat. 220, 144–155. 10.1111/j.1469-7580.2011.01461.x, PMID: 22150089 PMC3275769

[ref58] Waugh C. M. Korff T. Fath F. Blazevich A. J. (2014). Effects of resistance training on tendon mechanical properties and rapid force production in prepubertal children. J. Appl. Physiol. 117, 257–266. 10.1152/japplphysiol.00325.2014, PMID: 24903920 PMC4122689

[ref59] Zhang C. Couppé C. Scheijen J. L. J. M. Schalkwijk C. G. Kjaer M. Magnusson S. P.. (2020). Regional collagen turnover and composition of the human patellar tendon. J. Appl. Physiol. 128, 884–891. 10.1152/japplphysiol.00030.2020, PMID: 32163333

[ref60] Zwerver J. Bredeweg S. W. van den Akker-Scheek I. (2011). Prevalence of Jumper’s knee among nonelite athletes from different sports: a cross-sectional survey. Am. J. Sports Med. 39, 1984–1988. 10.1177/0363546511413370, PMID: 21737835

